# Supplementary material for: A Slam-dependent hemophore contributes to heme acquisition in the bacterial pathogen Acinetobacter baumannii
Source: Nat Commun. 2021 Nov 1;12:6270. doi: 10.1038/s41467-021-26545-9 (PMC8560813; doi:10.1038/s41467-021-26545-9)
Supplement: Supplementary file 1 — Supplementary Information [file 41467_2021_26545_MOESM1_ESM.pdf]

Supplementary Information:

A Slam-dependent hemophore contributes to heme acquisition in the bacterial pathogen *Acinetobacter baumannii*

Thomas J. Bateman[1], Megha Shah[1], Timothy Pham Ho[1], Hyejin Esther Shin[1], Chuxi Pan[1], Greg Harris[2], Jamie E. Fegan[3], Epshita A. Islam[1], Sang Kyun Ahn[3], Yogesh Hooda[1], Scott D. Gray-Owen[3], Wangxue Chen[2], Trevor F. Moraes[1*]

There are 14 Supplementary Figures and 3 Tables associated with this manuscript.

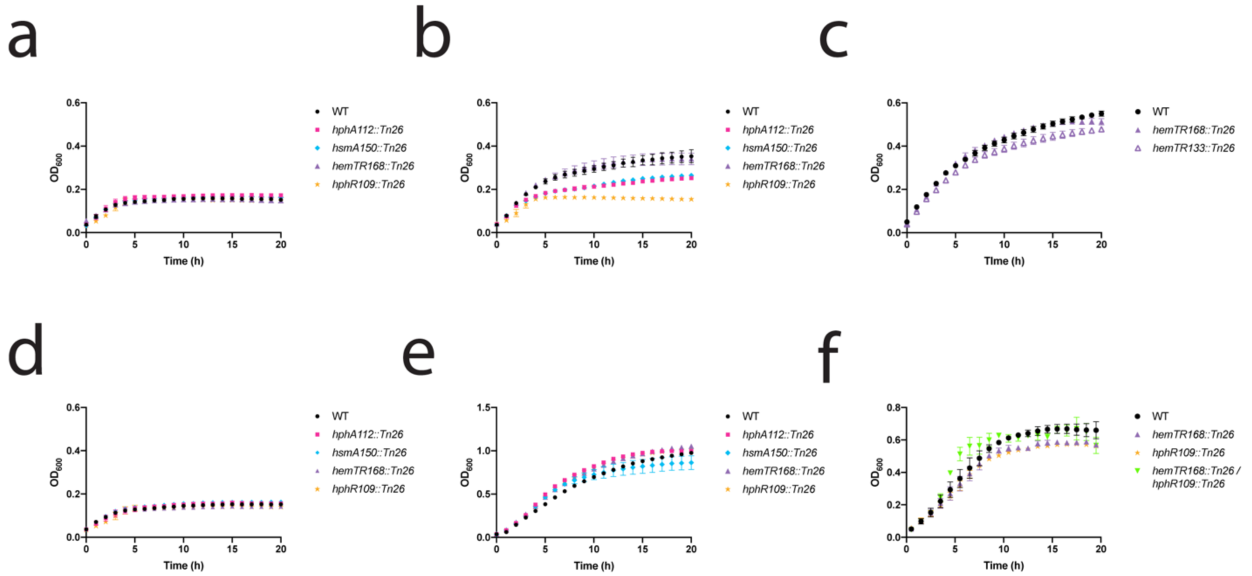

Supplementary Figure 1: *A. baumannii* growth curves.

a-d. Growth of iron starved WT and transposon mutant *A. baumannii* strains in RPMI (a), RPMI + 2 µM hemin (b), RPMI + 75 nM hemoglobin (c) or RPMI + 4 µM human serum albumin or HSA (d). Two independent HemTR transposon mutants were tested for a growth defect in the presence of RPMI supplemented with 75 nM hemoglobin. Growth curves for WT and *hemTR168::Tn26* are the same data as in Figure 1d. **e-f.** WT and *A. baumannii* mutants were grown in tryptic soy broth or TSB to check for general growth defects in iron rich media. Values plotted represent the mean ± standard error of the mean from three experiments.

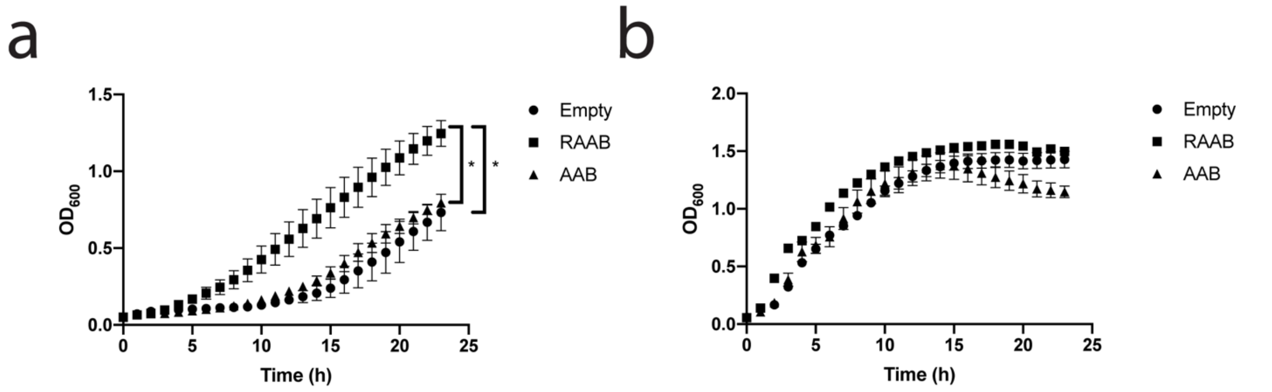

Supplementary Figure 2: Reconstitution of heme transport in *E. coli*.

a. Heme transport was reconstituted in *E. coli* by cloning the stretch of DNA encoding HphR-HphA-HsmA-TonB (RAAB) into an arabinose inducible expression vector (pHERD 20T), enabling growth in the presence of hemoglobin as a heme/iron source. Background growth likely from contaminating iron ions was observed in cells with empty vector or plasmid lacking HphR (AAB). Significance at the final time point determined by one-way ANOVA followed by Tukey's test. * $P<0.05$. Adjusted P values are as follows: Empty vs. RAAB P= 0.0124; Empty vs. AAB P= 0.8629; RAAB vs. AAB P= 0.0221. **b.** *E. coli* cells with empty pHERD vector, pHERD containing HphR-HphA-HsmA-TonB (RAAB), and pHERD HphA-HsmA-TonB (AAB) were grown in iron rich YT broth to determine if these genes enhance growth under normal growth conditions. Values plotted represent the mean ± standard error of the mean from three experiments.

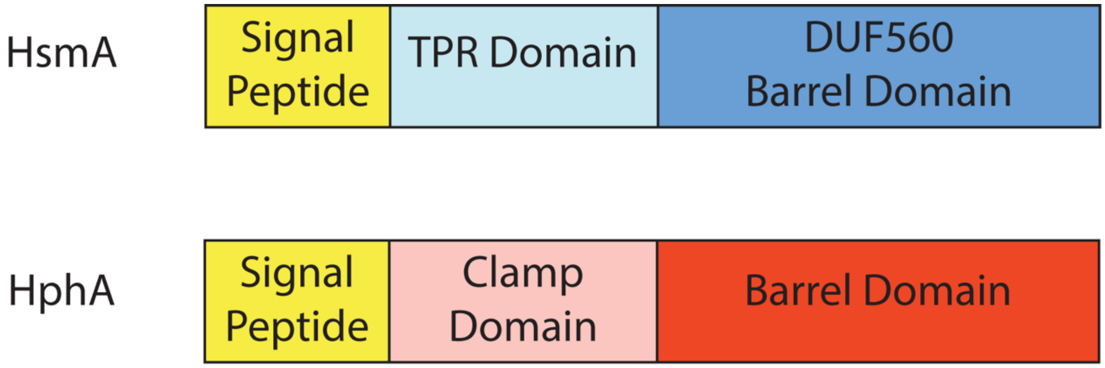

Supplementary Figure 3: Domain Architecture of HsmA and HphA.

HsmA is an outer membrane protein consisting of an N-terminal domain containing two tetratricopeptide repeats, and a C-terminal DUF560 domain predicted to be a 14 stranded β-barrel. HphA consists of an N-terminal clamp that contains a heme binding site, and a C-terminal 8 stranded β-barrel.

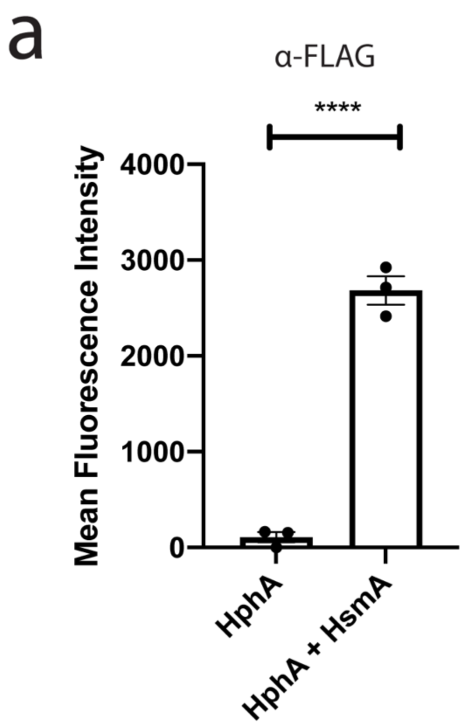

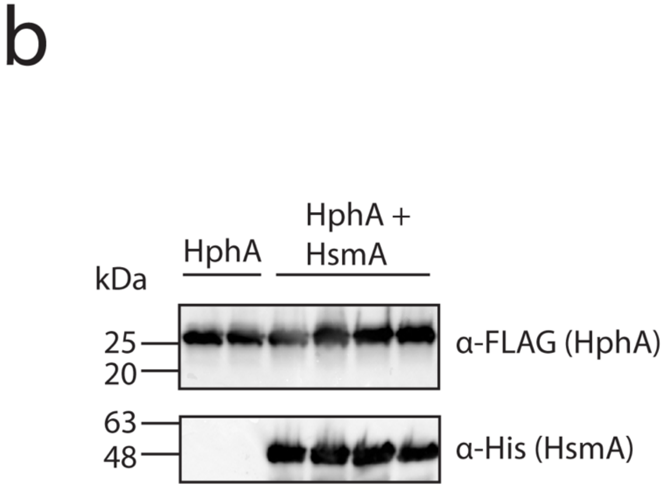

Supplementary Figure 4: HphA is a Slam dependent substrate.

a. An *E. coli* surface translocation assay was used to determine if HphA localizes to the bacterial cell surface, and whether this process is HsmA dependent. HphA's endogenous signal peptide was replaced with a pelB signal peptide followed by HphA's predicted lipobox motif. HphA and HsmA were expressed in *E. coli* C43 (DE3) cells. The cells were probed with rabbit α-FLAG followed by α-rabbit linked to R-phycoerythrin (R-PE) to detect HphA, and fluorescence intensity recorded by plate reader. Background fluorescence from cells stained with α-rabbit linked to R-PE was subtracted from plotted values and expressed as the mean fluorescence intensity ± standard error of the mean from three experiments. Data was analyzed using a two-tailed unpaired *t*-test. **** $P<0.0001$. **b.** Representative α-FLAG and α-His Western blots from three independent experiments confirming expression of HphA and HsmA respectively.

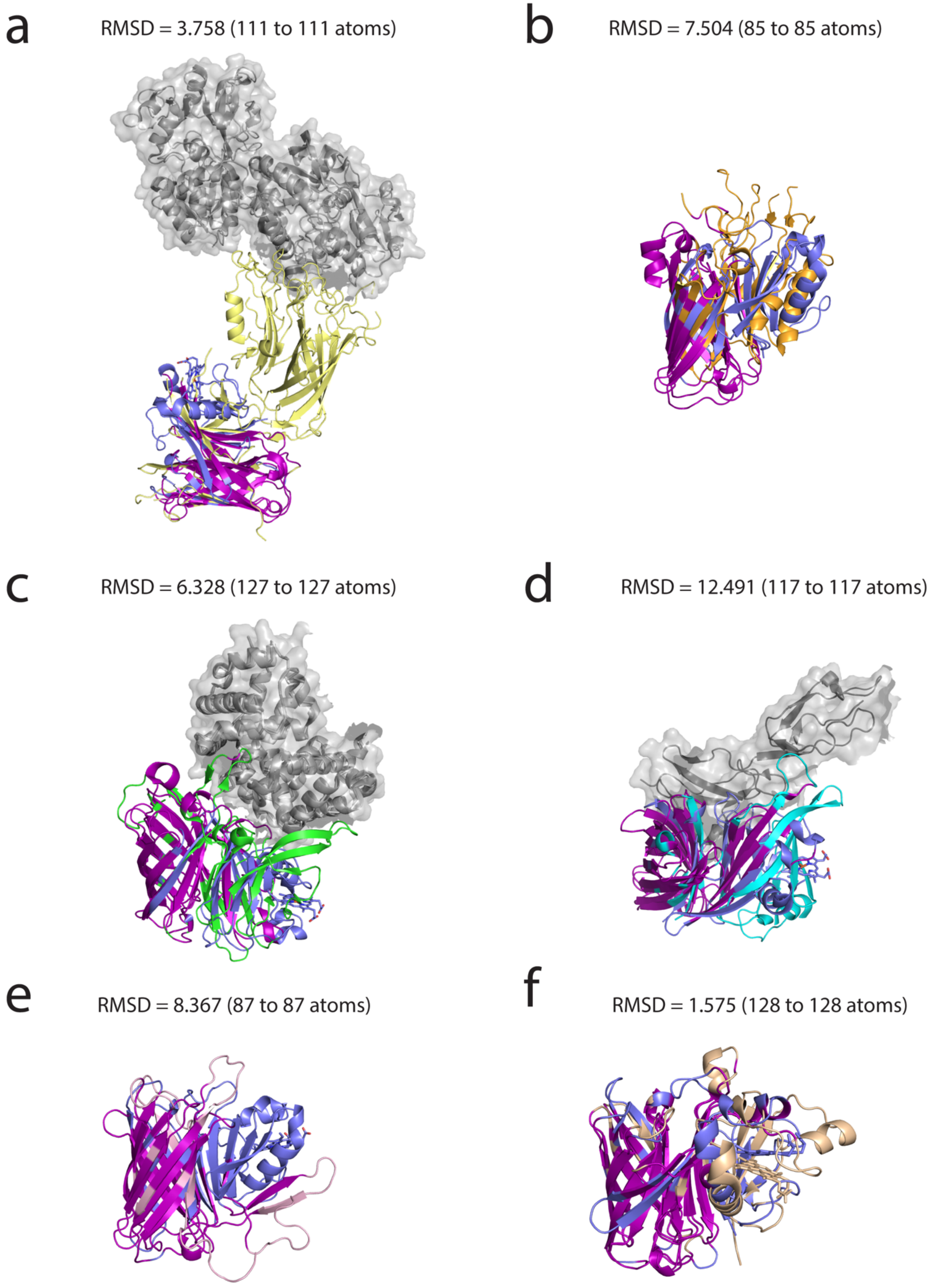
a
RMSD = 3.758 (111 to 111 atoms)
b
RMSD = 7.504 (85 to 85 atoms)
c
RMSD = 6.328 (127 to 127 atoms)
d
RMSD = 12.491 (117 to 117 atoms)
e
RMSD = 8.367 (87 to 87 atoms)
f
RMSD = 1.575 (128 to 128 atoms)

Supplementary Figure 5: Comparison of HphA to lipoprotein and hemophore structures. Structural superposition of HphA (blue) with the following five neisserial surface lipoproteins in PyMOL: **a.** Transferrin binding protein B (TbpB; yellow), **b.** Lactoferrin binding protein B (LbpB; orange), **c.** Hemoglobin-haptoglobin utilization protein A (HpuA; green), **d.** Factor H binding protein (fHBP; cyan) and **e.** Neisseria heparin binding antigen (NHBA; light pink). Neisserial SLP substrates (transferrin, hemoglobin and factor H) are colored gray. **f.** Also included is a structural alignment of HphA with the recently solved structure of a hemophore produced by *Haemophilus haemolyticus* (sand). Residues used in the alignment and calculation of the root-mean-square deviation (RMSD) via PyMOL are colored in magenta for each structural superposition.

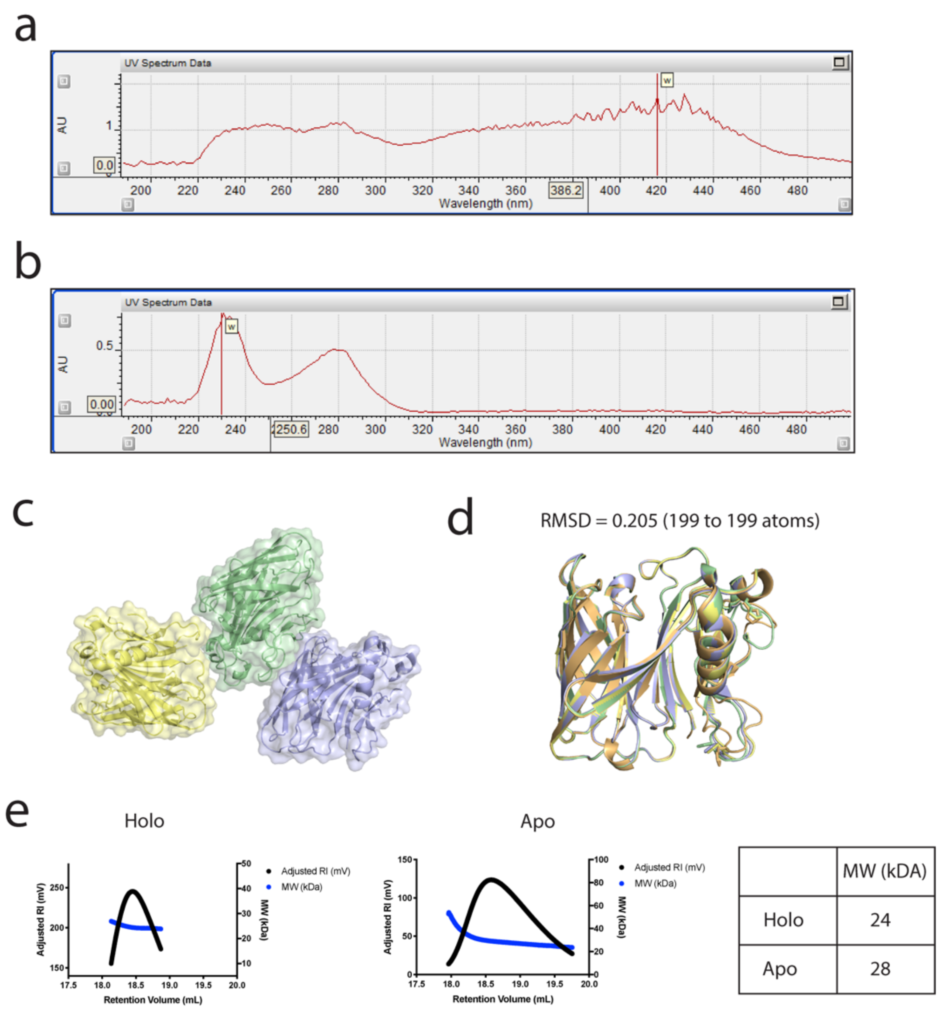

	MW (kDA)
Holo	24
Apo	28

Supplementary Figure 6: Comparison of holo and apo HphA heme content and oligomerization.

a-b. UV/Vis spectra data for holo (a) and apo (b) HphA. **c.** X-ray structure of apo HphA in the $C222_1$ space group. Each monomer chain is colored differently. **d.** Alignment of apo HphA in the $C222_1$ space group shown in blue, green and yellow with the apo protein in the $P2_12_12_1$ space group, depicted as a single monomer in orange. Heme coordinating His residues are depicted in stick form. **e.** Size exclusion chromatography and multi-angle light scattering analysis of holo and apo HphA.

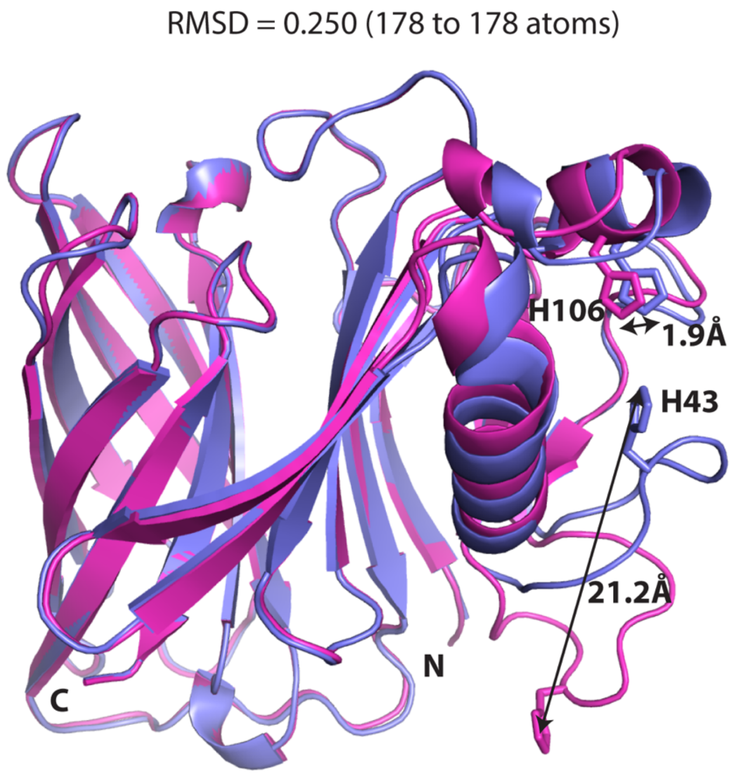

Supplementary Figure 7: Conformational changes in HphA upon binding and release of heme.

Alignment of the structures of holo (blue) and apo (magenta) HphA generated by PyMOL. His residues involved in heme coordination are shown in stick representation. Distances between equivalent residues in the apo and holo structures are indicated with arrows. The root-mean-square deviation (RMSD) is 0.250.

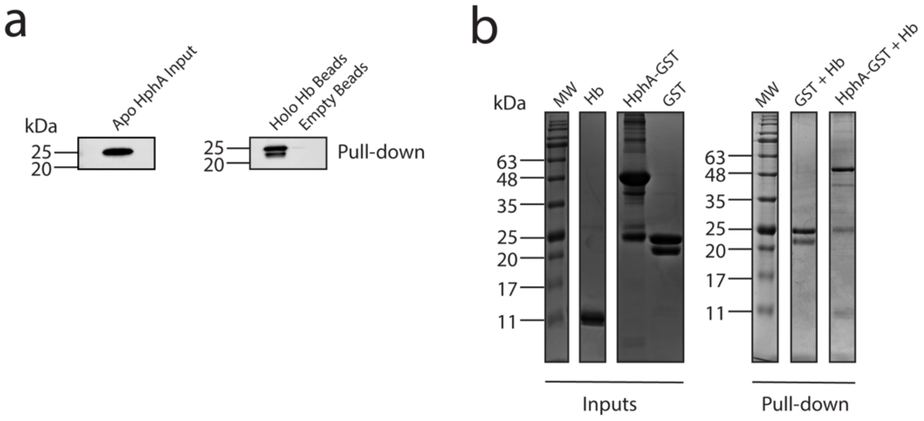

Supplementary Figure 8: Apo HphA binding is specific for hemoglobin.

a. Western blot showing that Apo HphA binds specifically to holo bovine hemoglobin (Hb) resin and not to empty beads. The input is one-tenth of the original concentration of apo HphA. The Western blots are representative of three independent experiments.

b. SDS-PAGE gels depicting the reciprocal experiment in which apo HphA-GST immobilized to glutathione resin pulled-down holo bovine Hb in solution. This interaction is not dependent on the tag as free GST did not pull-down Hb. Reciprocal pull-down was performed three independent times and representative gels are shown.

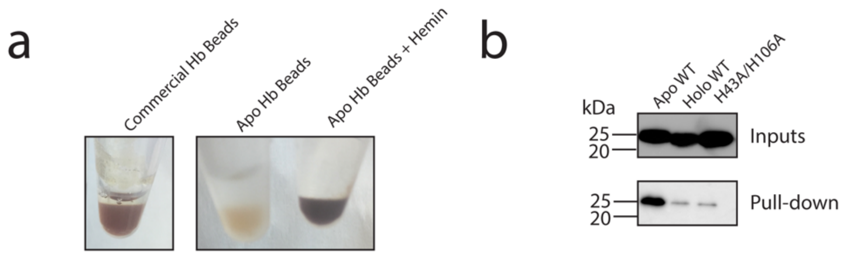

Supplementary Figure 9: Apo HphA interaction with hemoglobin is not dependent on hemoglobin's heme content.

a. Hemoglobin beads were treated with acid-acetone to remove heme, followed by refolding of apo hemoglobin. To assess functionality of refolded hemoglobin, apo beads were incubated with hemin and visually inspected for restoration of a reddish/brown color. **b.** Apo hemoglobin beads were incubated with apo, holo and H43A/H106A HphA. Binding of HphA to apo beads was assessed by Western blotting using α-His. Western blots are representative of three independent experiments.

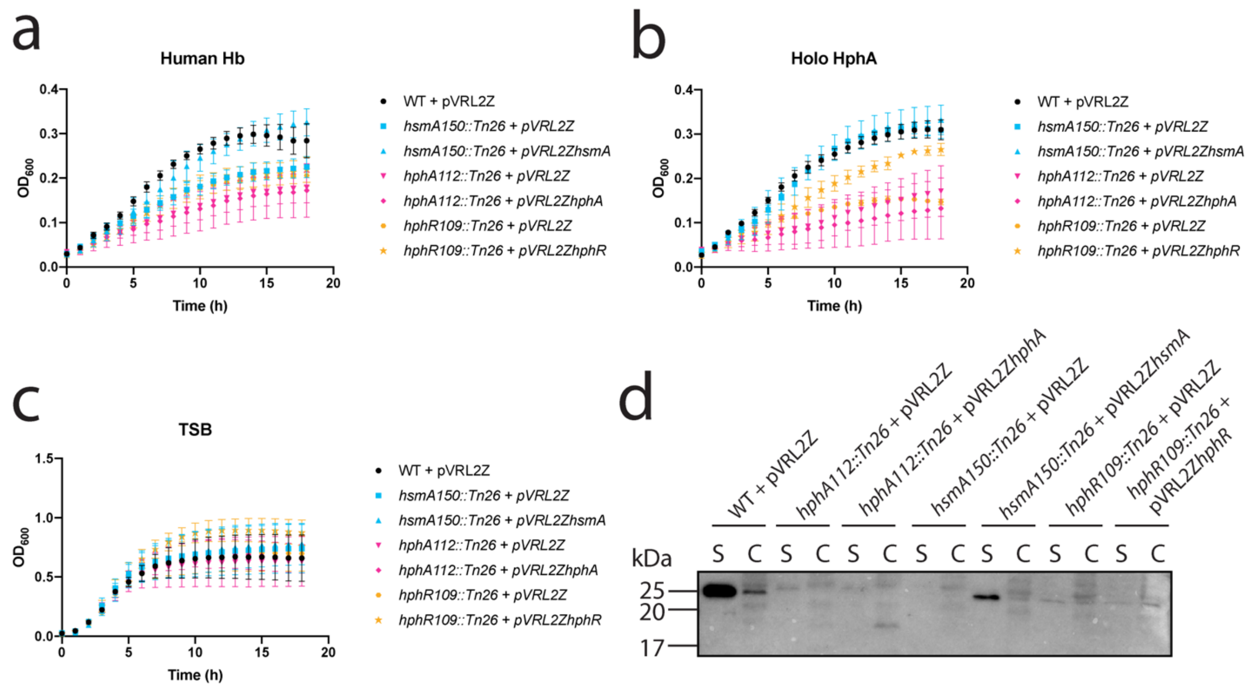

Supplementary Figure 10: Plasmid Complementation of *A. baumannii* transposon mutants.

a-c. *A. baumannii* strains transformed with empty pVRL2Z vector or vector containing HsmA, HphA or HphR genes were grown in low salt LB + 500 µg/mL Zeocin (Zeo) to OD_{600} of 0.6. 200 µM dipyridal (DIP) and 0.2% L-arabinose were added to restrict iron and induce protein expression respectively, and cells were switched to 20℃ growth for 20 h. Harvested cells were resuspended in RPMI with 75 nM human hemoglobin (a), 3 µM purified holo HphA (b) or tryptic soy broth (c). Growth at 37℃ was recorded over time. Values plotted represent the mean ± standard error of the mean from three experiments. **d.** HphA expression was assessed in transposon mutant and complemented strains by Western blot analysis of supernatant (S) and cell pellet (C) fractions. Expression analysis was performed once.

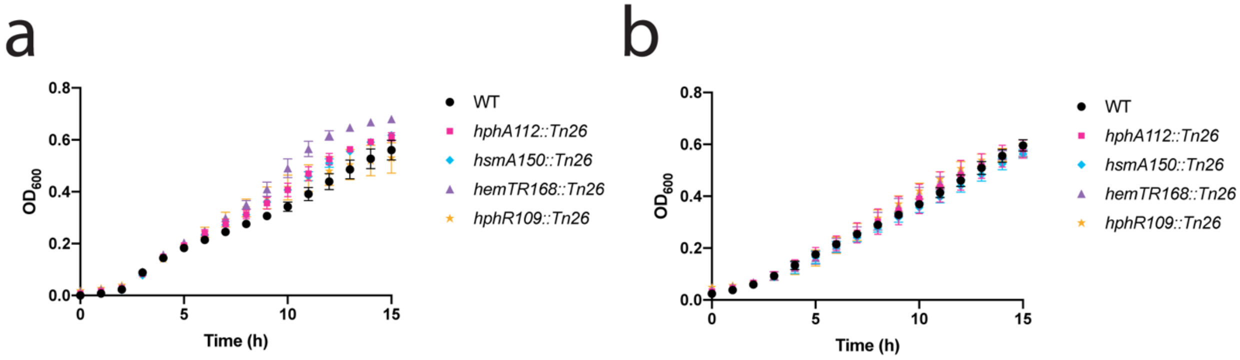

Supplementary Figure 11: Iron starved *A. baumannii* strains are not attenuated prior to being introduced into mice.

a. Frozen *A. baumannii* strains grown in LB containing 225 µM dipyridyl (DIP) as was done to starve the bacteria prior to sepsis challenge.

b. *A. baumannii* grown in conditions mimicking the ones used to grow the bacteria prior to mice pulmonary challenge. Briefly, *A. baumannii* was grown on chocolate agar plates supplemented with IsoVitaleX, resuspended in tryptic soy broth containing 225 µM DIP. All values plotted represent the mean ± standard error of the mean from three experiments.

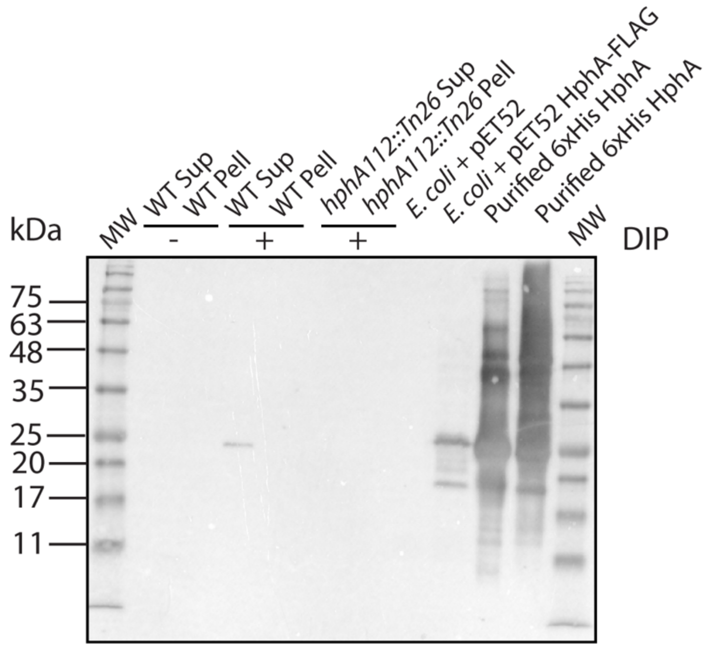

Supplementary Figure 12: HphA antibody validation.

The Western blot was obtained by probing the membrane with 1:2500 α-HphA followed by HRP linked goat α-mouse. HphA expression was induced by treating *A. baumannii* AB5075 with 200 µM dipyridyl (DIP). Antibody validation was performed once.

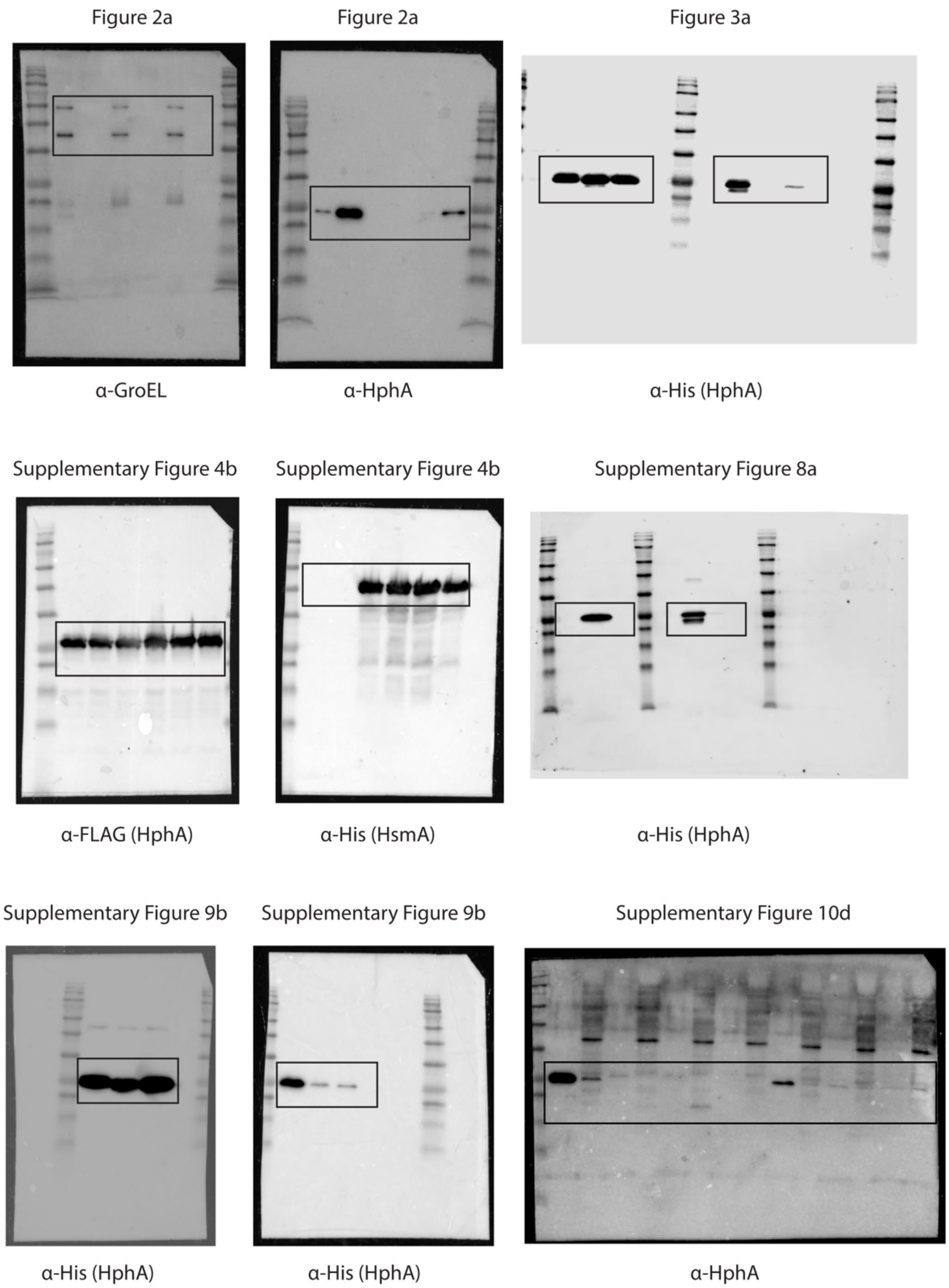

Supplementary Figure 13: Raw images for Western blots used in main and supplementary figures.

Lanes boxed in black are used in their respective figures.

Figure 3d

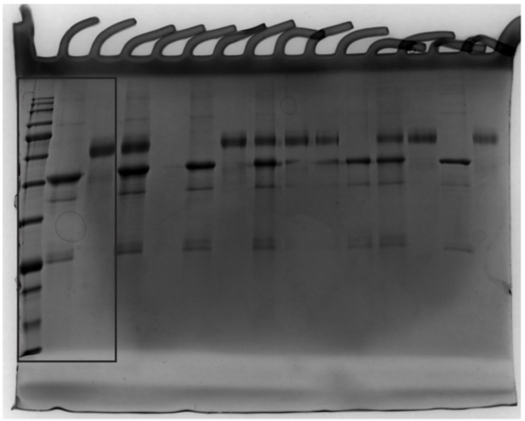

Supplementary Figure 8b Pull-down

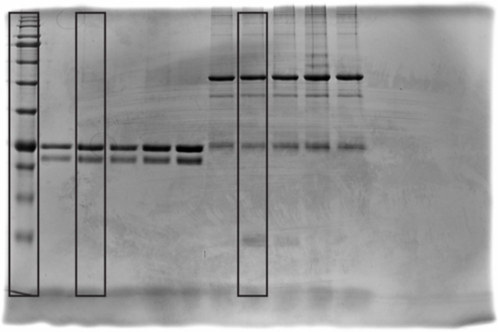

Supplementary Figure 8b Inputs

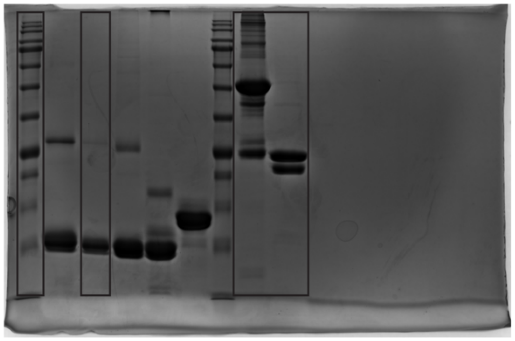

Supplementary Figure 14: Full SDS-PAGE gels from main and supplementary figures.

Lanes boxed in black are used in their respective figures.

	**Holo HphA**	**Apo HphA**	**Apo HphA**
PDB code	7RED	7REA	7RE4
Space group	$P2_12_12_1$	$P2_12_12_1$	$C222_1$
a, b, c (Å)	50.9 59.55 63.74	44.26 68.48 71.04	71.11 145.56 153.05
α, β, γ (°)	90 90 90	90 90 90	90 90 90
Wavelength (Å)	0.9796	0.9789	0.9789
Resolution (Å)	38.69 - 1.53 (1.58 - 1.53)	37.57 - 1.49 (1.54 - 1.49)	39.87 - 1.87 (1.937 - 1.87)
Total reflections	430705 (39814)	503481 (35505)	988494 (97596)
Unique reflections	29891 (2914)	35995 (3549)	65838 (6505)
Multiplicity	14.4 (13.7)	14.0 (10.0)	15.0 (15.0)
Completeness (%)	99.99 (99.97)	99.99 (99.89)	99.97 (99.95)
Mean I/σI	31.93 (9.01)	17.43 (4.29)	9.93 (1.67)
Wilson B-factor	10.09	9.15	19.15
R-merge	0.068 (0.388)	0.114 (0.533)	0.3014 (1.795)
R-meas	0.071 (0.403)	0.118 (0.562)	0.312 (1.858)
R-pim	0.018 (0.108)	0.031 (0.176)	0.08005 (0.4766)
CC1/2	1 (0.979)	0.999 (0.91)	0.995 (0.61)
CC*	1 (0.995)	1 (0.976)	0.999 (0.871)
Reflections used in refinement	29890 (2913)	35995 (3549)	65825 (6502)
Reflections used for R-free	1494 (146)	1800 (177)	3289 (324)
R-work	0.1496 (0.1665)	0.1470 (0.1774)	0.1723 (0.2783)
R-free	0.1684 (0.2087)	0.1694 (0.2077)	0.2093 (0.3323)
Number of non-hydrogen atoms	2020	2029	5530
Protein	1727	1707	5033
Ligands	43		
Solvent	250	322	497
Protein residues	243	244	722
RMS(bonds)	0.007	0.006	0.007
RMS(angles)	1.19	1.20	1.16
Ramachandran favored (%)	97.93	99.17	98.60
Ramachandran allowed (%)	2.07	0.83	1.26
Ramachandran outliers (%)	0.00	0.00	0.14
Rotamer outliers (%)	0.58	0.00	0.20
Clashscore	2.04	0.60	1.53
Average B-factor	12.20	12.29	22.82
Protein	10.50	9.87	22.11
Ligands	13.03		
Solvent	23.83	25.12	30.01

Supplementary Table 1 : Data collection and refinement statistics.

*Statistics for the highest-resolution shell are shown in parentheses.

Strain, Plasmid or Antibody	Genotype or Description	Reference
**Strains:**		
A. baumannii LAC-4	Hypervirulent strain isolated from a Los Angeles County Hospital in 1997. Belongs to the rare Pasteur MLST sequence type ST10. Classified as moderately antibiotic resistant. Sensitive to meropenem and imipenem, and intermediate sensitivity to amikacin. Complete genome sequence available.	[1]
A. baumannii AB5075	Hypervirulent strain isolated in 2008 from a Walter Reed Army Medical Center patient with osteomyelitis in the tibia. Pasteur MLST sequence type ST1. Sensitive to tetracycline, doxycycline, and high amounts of hygromycin and erythromycin. Available complete genome sequence and transposon mutant library make this a highly desirable strain for lab studies.	[2], [3]
A. baumannii AB5075 *hsmA150::Tn26* (tnab1_kr121205p02q150)	*ABUW_2983-150::Tn26*	[2]
A. baumannii AB5075 *hphA112::Tn26* (tnab1_kr121204p08q112)	*ABUW_2984-112::Tn26*	[2]
A. baumannii AB5075 *hemTR133::Tn26* (tnab1_kr130904p04q133)	*ABUW_2228-133::Tn26*	[2]
A. baumannii AB5075 *hemTR168::Tn26* (tnab1_kr130903p03q168)	*ABUW_2228-168::Tn26*	[2]
A. baumannii AB5075 *hphR109::Tn26* (tnab1_kr121204p05q109)	*ABUW_2985-109::Tn26*	[2]
A. baumannii AB5075 *hemTR168::Tn26*/ *hphR109::Tn26*	Double mutant created from *ABUW_2228-168::Tn26* and *ABUW_2985-109::Tn26*	This study
E. coli MM294	*F- glnX44(AS) LAM- rfbC1 endA1 spoT1 thiE1 hsdR17 creC510*	Yale CGSC #6315
E. coli C43 (DE3)	*F- ompT gal dcm hsdSB(rB- mB-)(DE3)*	[4]
E. coli BL21 Star (DE3)	*F- ompT hsdSB(rB- mB-) gal dcm rne131 (DE3)*	Invitrogen, C6010-03
SHuffle T7 Competent *E. coli*	F´ *lac*, *pro*, *lacI^q* / *Δ(ara-leu)7697 araD139 fhuA2 lacZ::T7 gene1 Δ(phoA)PvuII phoR ahpC* galE (or U) galK λatt*::pNEB3-r1-*cDsbC* (Spec^R, *lacI^q*) *ΔtrxB rpsL150*(Str^R) *Δgor Δ(malF)3*	New England BioLabs
E. coli JW0578-1	*F- Δ(araD-araB)567 ΔlacZ4787(::rrnB-3) ΔentF724::kan LAM- rph-1 Δ(rhaD-rhaB)568 hsdR514*	[5]
**Plasmids:**		
pET52b	*E. coli* expression vector	Novagen, 71554-3
pET52 HphA-FLAG	pET52b vector containing pelB signal peptide, *A. baumannii* LAC-4 HphA (residues 22-265/end), and C-terminal FLAG tag	This study
pET52 HphA-GST	pET52b vector containing *A. baumannii* LAC-4 HphA lacking its signal peptide (residues 22-265/end) followed by a PreScission protease cleavage site and GST tag.	This study
pET26b	*E. coli* expression vector	Addgene, 69862-3
pET26 His-HsmA	pET26b vector containing pelB signal peptide, 6XHis tag, and *A. baumannii* LAC-4 mature HsmA (residues 21-498/end)	This study
pET28a	*E. coli* expression vector	Novagen, 69864-3
pET28 His-HphA	pET28a vector containing N-terminal 6XHis tag and HphA lacking a signal peptide (residues 22-265/end)	This study
pET28 His-H43A/H106A-HphA	pET28a vector containing N-terminal 6XHis tag and mature HphA lacking a signal peptide (residues 22-265/end)	This study
pHERD20T	pBAD-based shuttle vector	[6]
pHERD RAAB	pBAD20T containing HphR, HphA, HsmA, and TonB related protein from *A. baumannii* LAC-4 (HphR cloned with 6XHis tag following its endogenous signal peptide)	This study
pHERD AAB	pBAD20T containing HphA, HsmA, and TonB related protein from *A. baumannii* LAC-4	This study

pVRL2Z	*A. baumannii* expression vector	[7]
pvRL2ZHsmA	pVRL2Z containing full length HsmA from strain AB5075, including endogenous signal peptide	This study
pvRL2ZHphA	pVRL2Z containing full length HphA from strain AB5075, including endogenous signal peptide	This study
pvRL2ZHphR	pVRL2Z containing full length HphR from strain AB5075, including endogenous signal peptide	This study
pABCre	Rifampicin selectable plasmid for expression of Cre Recombinase in *A. baumannii*	[2], [8]
**Antibodies:**		
α-FLAG	Rabbit polyclonal antibodies for FLAG (DYKDDDDK) epitope	Invitrogen, PA1-984B
α-His	Mouse monoclonal antibodies for polyhistidine tag	Pierce, MA121315
α-HphA	Mouse polyclonal antibodies produced in-house against His tagged HphA	This study
α-GroEL	Rabbit polyclonal antibodies raised against *E. coli* GroEL were obtained from Dr. Walid Houry	[9], [10]
PE-rabbit IgG	Phycoerythrin Conjugated F(ab')2 affinity purified anti-rabbit IgG	Rockland Immunochemical, CA711-108-002
HRP-rabbit IgG	Goat anti-rabbit IgG antibody conjugated to horseradish peroxidase (HRP)	Cell Signaling, 7074S
HRP-mouse IgG	Goat anti-mouse IgG antibody conjugated to horseradish peroxidase (HRP)	Pierce, PI31430
IRDye 800CW anti-mouse	Polyclonal goat IRDye 800CW anti-mouse secondary antibody obtained from Dr. Haley Wyatt	Li-COR, 926-32210

Supplementary Table 2: Strains, plasmids and antibodies used in this study.

Primer Name	Sequence	Purpose
F-FlagLAC4HphA<br>R-FlagLAC4HphA<br><br>F-FLAGinsLAC4HphA<br>R-FLAGinsLAC4HphA	5’-GATGACGACAAGTAAACCGCTGAGCAATAACTAGC-3’<br>5’-GTCTTTGTAGTCAGCGGAATTTTTAGCACCGCCAAATGC-3’<br><br>5’-GATGACGACAAGTAAACCGCTGAGCAATAAC-3’<br>5’-GTCTTTGTAGTCAGCGGAATTTTTAGC-3’	Round the horn cloning for constructing pET52 HphA-FLAG
F-CtermGST<br>R-CtermGST	5’-AACTTTAAGAAGGAGATATACATATGGGAATTGATGGTATTTCGAGTAATGAAAG-3’<br>5’-CATGGGCCCCTGGAACAGAACTTCCAGATTTTTAGCACCGCCAAATGCA-3’	Restriction free (RF) cloning pET52 HphA-GST
F-p26nHis-LAC4Slam<br>R-p26-LAC4Slam	5’-CCATGGCCCATCATCACCATCACCACGACGAAGACACACAGCTTCG-3’<br>5’-CAAGCTTGTCGACGGAGCTCGAATTCCTAAAAACGATGCTCAAGCTTGAAG-3’	Restriction free (RF) cloning pET26 His-HsmA
F-p26NHisLAC4HphA<br>R-p26NHisLAC4HphA	5’-GCAGCCATCATCATCATCATCACAGCGGAATTGATGGTATTTCGAGTAATGAAAG-3’<br>5’-TGTCCACCAGTCATGCTAGCCATATGTTAATTTTTAGCACCGCCAAATGC-3’	Restriction free (RF) cloning pET28 His-HphA
F-H43AHphA<br>R-H43AHphA<br><br>F-H106AHphA<br>R-H106AHphA	5’-GCCAATGCAAGTGCTCCGGGTGGTGTAGCAGC-3’<br>5’-CCACCCGGAGCACTTGCATTGGCAGCAGCACC-3’<br><br>5’-CAGTGTCTGCAATGCCACCTTCAGCCAGTGCATTAGG-3’<br>5’-ACTGGCTGAAGGTGGCATTGCAGACACTGGAATATGTG-3’	QuikChange mutagenesis for H43A/H106A substitutions in HphA
F-HphR<br>R-HphR<br>R-pHERD<br><br>F-HphA<br>R-TonB<br>R-pHERDHphR<br><br>F-SPHphR<br>R-SPHphR<br><br>F-SPHphR-2<br>R-SPHphR-2<br><br>F-SPHisHphR<br>R-SPHisHphR	Step 1: Clone HphR into pHERD20T<br>5’-ATGGGTGGGCTTGTTGTACTTC-3’<br>5’-TCGACTCTAGAGGATCCCCGGGTACCTTGTTGTTACTCCATCTTTTTTAAAAC-3’<br>5’-GGGTATGTATATCTCCTTCTTAAAGT-3’<br><br>Step 2: Clone HphA-HsmA-TonB into HphR-pHERD20T<br>5’-ATGAAAATCTCTCAATTGTTCTTGG-3’<br>5’-TCGACTCTAGAGGATCCCCGGGTACCTTAACGGCCCCGATTGAG-3’<br>5’-TTGTTGTTACTCCATCTTTTTTAAAAC-3’<br><br>Step 3: Clone endogenous HphR signal peptide and His tag into RAAB<br>5’-ATTTGCCATACATGCTGTGTCGATGGGTGGGCTTGTTGTACTTC-3’<br>5’-GCCAATGTTGAGAATCGAAAAAGGGTATGTATATCTCCTTCTTAAAG-3’<br><br>5’-GGCAAAAGCGGAATCGATCGATTTTTCGATTCTCAACATTGGCAT-3’<br>5’-CCGTTCCCGCTTTTCTCATGGGTATGTATATCTCCTTCTTAAAG-3’<br><br>5’-CATCACCACGAAACAGCCGGAGTTAAAAGC-3’<br>5’-GTGATGATGTGCATAAGCCTGCTGCATAG-3’	Exponential megapriming (EMP) cloning His-HphR-HphA-HsmA-TonB into pHERD20T (RAAB)
F-delHphR<br>R-delHphR	5’-ATGAAAATCTCTCAATTGTTCTTGG-3’<br>5’-GGGTATGTATATCTCCTTCTTAAAGTT-3’	Remove HphR from pHERD RAAB to generate pHERD AAB
F-CompSlam<br>R-CompSlam	5’-ACTTGACTCGAGATGAAACGAACGCTTTTGTGC-3’<br>5’-ATTACAGCGGCCGCCTAAAAACGATGCTCAAGCTTGAAG-3’	XhoI and NotI cloning pvRL2ZHsmA
F-CompHphA<br>R-CompHphA	5’-ACTTGACTCGAGATGAAAATCTCTCAATTGTTCTTGGG-3’<br>5’-ATTACAGCGGCCGCTTAATTTTTAGCACCGCCAAATGC-3’	XhoI and NotI cloning pvRL2ZHphA
F-CompHphR<br>R-CompHphR	5’-ACTTGACTCGAGATGAAAAAAGTGGGAACGGGG-3’<br>5’-ATTACAGCGGCCGCTTAAAATTTAGTGGTCCAACTAATTTTC-3’	XhoI and NotI cloning pvRL2ZHphR
F-T26<br>R-HemTR_TM<br>R-HphR_TM	5’-TGAGCTTTTTAGCTCGACTAATCCAT-3’<br>5’-GGATCTGCGTAACGTGGGT-3’<br>5’-TGGTCCAACTAATTTTCATCGTTCG-3’	Verification of AB5075 double mutant *hemTR168::Tn26*/ *hphR109::Tn26*

Supplementary Table 3: Primers used in this study.

References:

1. Ou, H.-Y. *et al.* Complete genome sequence of hypervirulent and outbreak-associated *Acinetobacter baumannii* strain LAC-4: epidemiology, resistance genetic determinants and potential virulence factors. *Sci Rep* **5**, 8643 (2015).
2. Gallagher, L. A. *et al.* Resources for Genetic and Genomic Analysis of Emerging Pathogen *Acinetobacter baumannii*. *J. Bacteriol.* **197**, 2027–2035 (2015).
3. Jacobs, A. C. *et al.* AB5075, a Highly Virulent Isolate of *Acinetobacter baumannii*, as a Model Strain for the Evaluation of Pathogenesis and Antimicrobial Treatments. *mBio* **5**, (2014).
4. Wagner, S. *et al.* Tuning *Escherichia coli* for membrane protein overexpression. *PNAS* **105**, 14371–14376 (2008).
5. Baba, T. *et al.* Construction of *Escherichia coli* K-12 in-frame, single-gene knockout mutants: the Keio collection. *Mol Syst Biol* **2**, 2006.0008 (2006).
6. Qiu, D., Damron, F. H., Mima, T., Schweizer, H. P. & Yu, H. D. PBAD-Based Shuttle Vectors for Functional Analysis of Toxic and Highly Regulated Genes in Pseudomonas and Burkholderia spp. and Other Bacteria. *Appl Environ Microbiol* **74**, 7422–7426 (2008).
7. Lucidi, M. *et al.* New shuttle vectors for gene cloning and expression in multidrug-resistant *Acinetobacter* species. *Antimicrob. Agents Chemother.* AAC.02480-17 (2018).
8. Gallagher, L. A., Lee, S. A. & Manoil, C. Importance of Core Genome Functions for an Extreme Antibiotic Resistance Trait. *mBio* **8**, e01655-17 (2017).

9. Houry, W., Frishman, D., Eckerskorn, C. *et al.* Identification of *in vivo* substrates of the chaperonin GroEL. *Nature* **402,** 147–154 (1999).

10. Ewalt,K. L., Hendrick,J. P., Houry,W. A. & Hartl,F. U. *In vivo* observation of polypeptide flux through the bacterial chaperonin system. *Cell* **90**, 491–500 (1997).
