# Supplementary material for: A Slam-dependent hemophore contributes to heme acquisition in the bacterial pathogen Acinetobacter baumannii
Source: Nat Commun. 2021 Nov 1;12:6270. doi: 10.1038/s41467-021-26545-9 (PMC8560813; doi:10.1038/s41467-021-26545-9)
Supplement: Supplementary file 3 — Reporting Summary [file 41467_2021_26545_MOESM3_ESM.pdf]

nature portfolio

Corresponding author(s): Trevor Moraes

Last updated by author(s): Sep 19, 2021

Reporting Summary

Nature Portfolio wishes to improve the reproducibility of the work that we publish. This form provides structure for consistency and transparency in reporting. For further information on Nature Portfolio policies, see our Editorial Policies and the Editorial Policy Checklist.

Statistics

For all statistical analyses, confirm that the following items are present in the figure legend, table legend, main text, or Methods section.

n/a	Confirmed	
☐	☒	The exact sample size (*n*) for each experimental group/condition, given as a discrete number and unit of measurement
☐	☒	A statement on whether measurements were taken from distinct samples or whether the same sample was measured repeatedly
☐	☒	The statistical test(s) used AND whether they are one- or two-sided<br>*Only common tests should be described solely by name; describe more complex techniques in the Methods section.*
☒	☐	A description of all covariates tested
☒	☐	A description of any assumptions or corrections, such as tests of normality and adjustment for multiple comparisons
☐	☒	A full description of the statistical parameters including central tendency (e.g. means) or other basic estimates (e.g. regression coefficient) AND variation (e.g. standard deviation) or associated estimates of uncertainty (e.g. confidence intervals)
☐	☒	For null hypothesis testing, the test statistic (e.g. *F*, *t*, *r*) with confidence intervals, effect sizes, degrees of freedom and *P* value noted<br>*Give P values as exact values whenever suitable.*
☒	☐	For Bayesian analysis, information on the choice of priors and Markov chain Monte Carlo settings
☒	☐	For hierarchical and complex designs, identification of the appropriate level for tests and full reporting of outcomes
☒	☐	Estimates of effect sizes (e.g. Cohen's *d*, Pearson's *r*), indicating how they were calculated

Our web collection on statistics for biologists contains articles on many of the points above.

Software and code

Policy information about availability of computer code

Data collection	XDS package version Oct 15,2015
Data analysis	GraphPad Prism (9.0.0), Coot (0.8.9), Phenix Program Suite (1.13_2998), PyMOL (1.8.6.0)

For manuscripts utilizing custom algorithms or software that are central to the research but not yet described in published literature, software must be made available to editors and reviewers. We strongly encourage code deposition in a community repository (e.g. GitHub). See the Nature Portfolio guidelines for submitting code & software for further information.

Data

Policy information about availability of data

All manuscripts must include a data availability statement. This statement should provide the following information, where applicable:

- Accession codes, unique identifiers, or web links for publicly available datasets
- A description of any restrictions on data availability
- For clinical datasets or third party data, please ensure that the statement adheres to our policy

X-ray data (atomic coordinates and structure factors) has been deposited in the RCSB PDB with the following accession codes: holo HphA (7RED) [https://doi.org/10.2210/pdb7RED/pdb], apo HphA – P212121 form (7REA) [https://doi.org/10.2210/pdb7REA/pdb], and apo HphA – C2221 form (7RE4) [https://doi.org/10.2210/pdb7RE4/pdb].
All other source data are provided with this paper.

Field-specific reporting

Please select the one below that is the best fit for your research. If you are not sure, read the appropriate sections before making your selection.

☒ Life sciences ☐ Behavioural & social sciences ☐ Ecological, evolutionary & environmental sciences

For a reference copy of the document with all sections, see nature.com/documents/nr-reporting-summary-flat.pdf

Life sciences study design

All studies must disclose on these points even when the disclosure is negative.

Sample size

For the pulmonary challenge, the number of mice for each group was empirically estimated based on the published literature on similar types of studies and the collective experiences of the investigators and the results from similar studies (Antimicrobial agents and chemotherapy. 2013;57(8):3601-13; Sci Rep. 2019;9(1):6538). 5 mice per group were used in all experiments so that the number of animals was kept at a minimal while at the same time a statistically meaningful result could be obtained.

For the sepsis challenge, based on the results of a pilot dose-ranging study and our experience with sepsis models in general, we determined that a sample size of 6 would be sufficient to achieve partial lethality with the WT strain and to evaluate an effect with the mutants.

Data exclusions

No data was excluded from the analyses.

Replication

Experiments were repeated 3 times to ensure reproducibility with the exceptions of Supplementary Figure 6e SEC-MALS data, Supplementary Figure 10d, which was performed once as additional validation of Supplementary Figure 10 a-c, and Supplementary Figure 12 for antibody validation. All attempts at replication were successful.

Randomization

For the pulmonary challenge, mice were randomly assigned to each infection group on a cage basis. Our experience with mouse models of A. baumannii infection including this strain showed that randomization of individual mice from different cages is unnecessary and co-housing of mice from different cages after randomization put additional stress on the animals.

For the sepsis challenge, we utilized inbred mice of the same age and weight range, which were co-housed and kept under specific-pathogen free conditions. Since there was little variability in genetics or the microbiome of these animals, we did not expect there to be much variability in their response to infection. Mice were randomized into cages upon receipt from the vendor (Charles River) and then subsequently separated into different treatment groups.

Blinding

Blinding was not relevant to this study as objective measures such as mice survival were reported.

Reporting for specific materials, systems and methods

We require information from authors about some types of materials, experimental systems and methods used in many studies. Here, indicate whether each material, system or method listed is relevant to your study. If you are not sure if a list item applies to your research, read the appropriate section before selecting a response.

Materials & experimental systems

n/a	Involved in the study	
☐	☒	Antibodies
☒	☐	Eukaryotic cell lines
☒	☐	Palaeontology and archaeology
☐	☒	Animals and other organisms
☒	☐	Human research participants
☒	☐	Clinical data
☒	☐	Dual use research of concern

Methods

n/a	Involved in the study	
☒	☐	ChIP-seq
☒	☐	Flow cytometry
☒	☐	MRI-based neuroimaging

Antibodies

Antibodies used

Commercially available antibodies used in this study include: anti-FLAG (Invitrogen, PA1-984B), anti-His (Pierce, MA121315), PE conjugated rabbit IgG (Rockland Immunochemical CA711-108-002), HRP conjugated rabbit IgG (Cell Signalling 7074S), HRP conjugated mouse IgG (Pierce, PI131430), IRDye 800CW anti-mouse (Li-COR, 926-32210). Mouse anti-HphA was made in-house, and anti-GroEL obtained from Dr. Walid Houry (University of Toronto).

Validation

Validation of the following primary antibodies for the species and application (Western Blots) can be found on the manufacturer's website: His (https://www.thermofisher.com/antibody/product/6x-His-Tag-Antibody-clone-HIS-H8-Monoclonal/MA1-21315), and FLAG (https://www.thermofisher.com/antibody/product/DYKDDDDK-Tag-Antibody-Polyclonal/PA1-984B). FLAG has also been used for cell surface staining (relevant citation: Hooda, Yogesh et al. Identification of a Large Family of Slam-Dependent Surface Lipoproteins in Gram-Negative Bacteria. Frontiers in cellular and infection microbiology vol. 7, 207 (2017).)

Mouse anti-HphA was validated for species and application (Western Blots) using various bacterial strains including A. baumannii transposon knockout and E. coli expressing exogenous HphA (Supplementary Figure 12).
Anti-GroEL antibodies raised against E.coli GroEL were obtained from Dr. Walid Houry, and previously validated for species (E.coli) and application (Western Blots), but used in this study for detection in A. baumannii. Relevant citations:
1. Houry, W., Frishman, D., Eckerskorn, C. et al. Identification of in vivo substrates of the chaperonin GroEL. Nature 402, 147–154 (1999).
2. Ewalt,K. L., Hendrick,J. P., Houry,W. A. & Hartl,F. U. In vivo observation of polypeptide flux through the bacterial chaperonin system. Cell 90, 491–500 (1997).
3. Hooda, Yogesh et al. Identification of a Large Family of Slam-Dependent Surface Lipoproteins in Gram-Negative Bacteria. Frontiers in cellular and infection microbiology vol. 7, 207 (2017).

Animals and other organisms

Policy information about studies involving animals; ARRIVE guidelines recommended for reporting animal research

Laboratory animals	For the pulmonary challenge, 6 to 10 weeks-old specific-pathogen-free, female BALB/c mice were used.<br>For the sepsis challenge, 8 to 10 weeks-old specific-pathogen-free, male C57BL/6 mice were used.
Wild animals	No wild animals were used in the study.
Field-collected samples	No field-collected samples were used in the study.
Ethics oversight	For the pulmonary challenge, experimental procedures were approved by the institutional animal care committee (AUP#2016.11, Human Health Therapeutics Research Center, National Research Council Canada, Ottawa). For the sepsis challenge, experiments were done in accordance with University of Toronto Animal Ethics Review Committee under protocol 20011319.

Note that full information on the approval of the study protocol must also be provided in the manuscript.
